# HSP-70 and TNF-α as predictors of acute respiratory distress syndrome in children with pneumonia

**DOI:** 10.1186/s12887-025-06297-x

**Published:** 2025-12-12

**Authors:** Hisham W. Bader, Rehab S. I. Moustafa, Mohamed A. Shahba, Marwa Elhady, Mohamed Sobhy Mansour, Moushira Zaki, Hanaa Reyad Abdallah, Mina Wassef Girgiss, Eman Refaat Youness

**Affiliations:** 1 Child Health Department, Medical Research and Clinical Studies Institute, National Research Centre, Cairo, Egypt; 2 Medical Biochemistry Department, Medical Research and Clinical Studies Institute – National Research Centre Cairo, Cairo, Egypt; 3 Pediatric Department, Faculty of Medicine for Girls, Al-Azhar University, Cairo, Egypt; 4 Pediatric Department, Faculty of Medicine for Boys, Al-Azhar University, Cairo, Egypt; 5 Biological Anthropology Department, Medical Research and Clinical Studies Institute–National Research Centre Cairo, Cairo, Egypt; 6 Medical Research and Clinical Studies Institute–National Research Centre Cairo, Cairo, Egypt

**Keywords:** ARDS, Pneumonia, Heat shock protein, TNF-α

## Abstract

Community acquired pneumonia is a leading cause of pediatric hospitalization worldwide. Identifying predictor for pneumonia severity and complications is critical to improve the diagnosis and outcome. This study aimed to evaluate the role Heat shock protein-70 (HSP-70) and Tumor necrosis factor alpha (TNF-α) as predictors of acute respiratory distress syndrome (ARDS) in children with pneumonia. The current cross sectional study included 60 child with pneumonia who were referred for admission in pediatric intensive care during the period from February 2024 to July 2024. Clinical data, HSP-70, TNF-α serum levels, isolated bacteria and bacterial biofilm formation were assessed in all children. We found that 15 out of 60 included children developed ARDS (25%). Heat shock protein-70 and TNF-α were statistically significant higher in those who developed ARDS than those without ARDS. Isolated bacteria and a higher rate of biofilm formation were substantially higher in patients who developed ARDS. HSP-70 had 93.3% sensitivity and 91.1% specificity at cutoff point of 5.5 ng/ml and TNF-α had 93.3% sensitivity and 86.7% specificity at a cutoff point of 442 pg/ml to predict ARDS in children with pneumonia. Elevated HSP-70 (OR:10.5) elevated TNF- α (OR: 6), bacterial biofilm formation (OR:7.3), respiratory acidosis (OR:6), and low PaO2/FiO2 < 300 (OR:1.667) were independent predictors for development of ARDS. In conclusion, several confounders contribute to the development of ARDS in children with pneumonia included inflammatory biomarkers, bacterial biofilm formation, respiratory acidosis, and reduced PaO₂/FiO₂ ratios. HSP-70 and TNF-α showed good predictive performance, indicating their potential utility as early biomarkers for identifying high-risk patients.

## Introduction

 Pneumonia is a common pediatric infectious disease and a principal cause of hospitalization and death in children under five years worldwide. Pneumonia etiological distribution differs widely by epidemiological season of respiratory pathogens and age [1]. Pneumonia induced direct alveolar epithelial and capillary endothelial injury that triggers the development of acute respiratory distress syndrome (ARDS) [2]. Mortality rates increased in low- and middle-income countries and associated with a significant financial burden [3]. Whereas pneumonia and death rates have significantly reduced, hospitalization rates and hospital visits are increasing in both developed and developing countries [4].

Heat shock protein-70 (HSP-70) is the most adaptable chaperone protein responsible for the folding of proteins produced within the cell. This protein aids in refolding partially denatured proteins caused by various stresses, such as shock and infections [5]. In ARDS, HSP-70 contributes to both external pro-inflammatory actions and intracellular cytoprotection, which may appear contradictory. In the context of ARDS, HSP-70 interferes with apoptotic pathways by binding to apoptotic protease and preventing caspase activation, thereby reducing alveolar epithelial and endothelial cell death. Additionally, it lessens cellular damage caused by reactive oxygen species, a major factor in the pathophysiology of ARDS [6]. HSP-70 reduces the expression of key inflammatory mediators and has an anti-inflammatory effect. Paradoxically, when HSP-70 is released into the extracellular space, it can also make inflammation worse. HSP-70 binds to TLR2/4 and CD14, which activates NF-KB and releases pro-inflammatory cytokines such TNF-α, IL-1B, and IL-6 [7].

TNF-α is a pro-inflammatory cytokine. In bacterial pneumonia macrophage-derived TNF-α is elevated, resulting in the recruitment of inflammatory cells to sites of infection. On the other hand, TNF-α may not show a significant antiviral role, and its serum level does not change significantly during viral pneumonia [8, 9].

This study aimed to evaluate the role HSP-70 and TNF-α as predictors of ARDS in children with pneumonia with correlation of clinical data, biological, and inflammatory biomarkers.

## Subjects and methods

The current cross-sectional study with a control group included 60 children diagnosed with pneumonia who were referred for admission in pediatric intensive care at Al-Azhar University Hospitals during the period from February 2024 to July 2024. Fifty healthy, sex- and age-matched subjects were enrolled as the control group. Children with pneumonia were divided into two groups: group 1 included pneumonia complicated by ARDS, and group 2 involved pneumonia without ARDS.

### Ethical approval

The study was authorized by the Al-Azhar University Faculty of Medicine's Ethical Committee (No. 000189/2024), in accordance with the World Medical Association's Declaration of Helsinki. 

 After the parents or guardians of the children were informed about the study, they provided written informed consent to participate.

### Inclusion criteria

Children and infants of both sexes aged 4 months to 36 months, diagnosed with pneumonia were enrolled in the study after their caregivers approved the study and gave written consent.

### Exclusion criteria

Subjects with respiratory illnesses other than pneumonia, as well as those with underlying cardiac, hepatic, renal, or neurological disorders, were excluded from the study.

ARDS was diagnosed based on the definition recommended by the Second Pediatric Acute Lung Injury Consensus Conference (PALICC-2) guidelines published in 2023. Children who develop acute respiratory failure within 7 days after clinical insult that is not explained by heart failure, fluid overload or perinatal pathology with radiological new opacities caused by acute pulmonary parenchymal disease that is not caused by atelectasis or pleural effusion together with oxygenation index (OI) ≥ 4 or oxygenation saturation index (OSI) ≥ 5 in ventilated children or Pao2/FiO2 ≤ 300 or SpO2/FiO2 ≤ 250 in non-invasive ventilated children [10].

### Procedure

All included subjects underwent detailed history taking for presenting respiratory manifestations, onset and duration of current illness, symptoms suggestive of other systems' affection, and complete local respiratory and systemic examination, including vital data on admission.

Anthropometric measurements were taken using standardized equipment and weight and height Z-scores were calculated based on WHO growth charts specific for age and sex.

Laboratory investigations on admission included arterial blood gases, complete blood count with differential leucocytic count, acute phase reactants (ESR and CRP), serum electrolytes, renal and liver function tests.

Quantification of HSP-70was estimated using ELISA kit, and the serum concentrations of HSP-70 were measured twice according to NOVA (Bioneovan, Beijing, China).

Quantification of TNF- α was estimated using ELISA kit according SinogeneClon Biotech Co. Catalog No. SL-1761 Hu.

Sputum cultures were obtained via tracheal aspirates in intubated patients and nasopharyngeal aspirate in non-ventilated patients. Collection was doen under aseptic technique using early morning samples to minimize contamination.

Biofilm detection was done using Congo red agar method which is a qualitative method detecting biofilm producing bacteria. 0.8 gm of Congo red dye was added to 1000 ml of distilled water and mixed well then autoclaved at 121°c for 15 min. Agar media was prepared by adding 37 g Brain heart infusion broth, 50 gm sucrose, 10 gm agar base to 1000 ml of distilled water and autoclaved at 121°c for 15 min. When media is cooled to 55°c the Congo red was added to the brain heart infusion agar. Congo red agar was placed in sterile plates. Isolated bacteria were inoculated on Congo red agar plates and then incubated at 35°c ± 2 for 24 hours. Black colonies with a dry crystalline consistency indicate strong biofilm production while Dark colonies with no dry crystalline indicate moderate biofilm production; whereas colonies remained pink color are considered non-biofilm producers [11].

### Statistical analysis

The data was analyzed using version 23.0 of the SPSS program. The normality of the variables was examined using the Shapiro-Wilk test. A one-way analysis of variance (ANOVA) was conducted for parametric data when comparing more than two means. Post Hoc tests, including Tukey's and Mann-Whitney U tests, were utilized for multiple comparisons between different variables. The comparison between groups with qualitative data was performed using the Chi-square test only when the expected count in any cell was less than 5. Receiver operating characteristic (ROC curve) analysis was used to identify the overall predictivity of parameters and to determine the appropriate cut-off values, including the detection of sensitivity and specificity at these values.

Multivariate logistic regression analysis: Odds ratios (OR) with 95% confidence intervals were conducted to assess potential risk factors. The confidence interval was 95%, and the acceptable margin of error was 5%. Thus, P-values less than 0.05 were regarded as significant.

## Results

Of the 60 children included with pneumonia, 15 developed ARDS (25%). Control group included 28 male and 22 females with mean age of 15.86±3.42 months, body weight Z-score ranged between −0.5-1.7 with mean value of 0.46±0.47, their length z-score ranged between −1.4-1.5 with mean value of 0.10±0.84. There was statistically insignificant difference between patients and control group as regard age and sex (p-value 0.256, 0.569 respectively). Comparing baseline clinical data on admission revealed statistically significant higher heart rate and respiratory rate in those who later developed ARDS than those who did not develop ARDS while there was no significant difference as regard oxygen saturation, temperature, duration of ICU stay, number of previous pneumonia, number of previous hospital admission and type of pneumonia in X-ray between both groups. Subjects with ARDS have lower weight z-score than those without ARDS and healthy controls while there was statistically insignificant difference between weight Z-score between those who did not develop ARDS and healthy control. Height z-score measurements show statistically insignificant difference between the studied groups.

Regarding the laboratory investigations on admission, subjects who developed ARDS had statistically significant higher liver enzymes (ALT & AST), lower serum Na level, and lower PaO2/FiO2 ratio than those who did not develop ARDS, suggesting a ventilation perfusion mismatch.

Furthermore, acute phase reactants (ESR and CRP) on admission together with the serum levels of heat shock protein and TNF-a, were statistically significantly higher in those who developed ARDS than those who did not, as shown in Table 1.

**Table 1:** Comparison of clinical and radiological data of the studied population according to ARDS

		Pneumonia complicated by ARDS (*n* = 15)		Pneumonia without ARDS (*n* = 45)		Independent student t test/Mann Whitney U test/chi square test	
		Mean	SD	Mean	SD	z/t/x2	P-value
Male sex (N, %)		10 (66.7%)		27 (60%)		0.212	0.646
Age		16.93	7.81	17.80	9.85	−0.348	0.731
HR beat/min		119.80	7.29	114.13	7.47	2.591	0.016
RR		61.20	5.03	57.09	3.79	2.901	0.009
Temp (C)		38.74	1.30	38.20	1.11	1.457	0.160
O2sat (%)		93.00	1.31	92.24	2.06	1.655	0.106
Wt (Kg)		9.23	2.11	10.11	2.27	−1.383	0.179
Wt z-score		−1.01	0.85	0.32	0.82	−5.286	0.000
Ht (cm)		63.40	4.45	64.53	6.87	−0.736	0.466
Ht z-score		−0.13	0.70	0.04	1.08	−0.736	0.466
ICU stay		8.73	0.46	8.38	1.19	1.666	0.101
X-ray	Lobar pneumonia	6 (40%)		12 (26.7%)		0.952	0.329
	Bronchopneumonia	9 (60%)		33 (73.3%)			

Sputum culture revealed statistically more isolated bacteria and a higher rate of biofilm formation in subjects who developed ARDS, as shown in Table 2.

**Table 2:** Comparison of hematological and biochemical data of the studied population according to ARDS

	Pneumonia complicated by ARDS (*n* = 15)		Pneumonia without ARDS (*n* = 45)		Independent student t test/Mann Whitney U test/chi square test	
	Mean	SD	Mean	SD	z/t/x2	P-value
HB (g/dL)	10.03	0.85	10.42	1.11	−1.450	0.157
HCT	30.14	2.33	31.25	3.34	−1.421	0.164
WBCs	8.95	1.88	9.17	2.15	−0.381	0.706
Platelets (mcL)	300.60	11.55	302.87	26.02	−0.463	0.645
AST (U/L)	40.53	24.04	22.93	8.81	2.774	0.014
ALT (U/L)	48.73	26.51	28.78	6.43	2.888	0.012
Urea (mg/dl)	26.40	1.84	27.89	15.54	−0.630	0.532
Creat (mg/dl)	0.70	0.07	0.75	0.32	−0.865	0.391
Na (mmol/L)	132.60	0.99	135.33	5.02	−3.456	0.001
K (mmol/L)	3.34	0.40	3.41	0.32	−0.594	0.559
Ca (mg/dl)	9.70	0.07	9.55	1.05	0.988	0.328
ESR (mm/hr)	57.13	13.26	43.78	14.10	3.325	0.003
CRP (mg/dl)	84.20	27.75	34.16	27.56	6.059	0.000
Glucose (mg/dl)	90.40	9.26	95.49	7.96	−1.906	0.070
PH	7.29	0.08	7.37	0.05	−3.470	0.003
PCO2 mm Hg	43.73	8.04	41.76	6.78	0.857	0.401
PO2mm Hg	63	23.68	76	28.49	0.693	0.499
HCO3 mEq/L	20.95	2.56	22.72	4.30	−1.926	0.061
PaO2/FiO2	126	22.91	304	85.99	−10.178	0.000

Independent student t, Mann Whitney U and chi square tests; *P* < 0.05 = significance

*ARDS* Acute respiratory distress syndrome, *SD* Standard deviation, *HR* Heart rate, *RR* Respiratory rate, *Temp* Temperature, *O2 sat* Oxygen saturation, *Wt* Weight, *Ht* Height, *BMI* Body mass index, *PICU* Pediatric intensive care unit, *Hb* Hemoglobin, *HCT* Hematocrit value, *WBCs* White blood cells, *AST* Aspartate transaminase, *ALT* Alanine transaminase, *Creat* Creatinine, *Na* Sodium, *K* Potassium, *Ca* Calcium, *CRP* C-reactive protein, *ESR* Erythrocytic sedimentation rate, *PO2* Partial pressure of Oxygen, *PCO2* Partial pressure of carbon dioxide, *HCO3* Bicarbonate, *PaO2/FiO2* Partial pressure of oxygen in arterial blood, fraction of inspired oxygen ratio

The current study showed statistically significant higher AST, and acute phase reactants and lower PH, HCO3, and PaO2/FiO2 in subjects who had isolated bacteria with biofilm formation, as shown in Table 3.

**Table 3:** Comparison of sputum culture findings among the studied population according to ARDS

		Pneumonia complicated by ARDS (*n* = 15)	Pneumonia without ARDS (*n* = 45)	Chi square test	
				X2	P-value
Organism (sputum culture)	Bacteria	11 (73.3%)	16 (35.6%)	6.890	0.032
	Fungal	1 (6.7%)	3 (6.7%)		
	No-growth	3 (20%)	26 (57.8%)		
Isolated bacteria	MRSA	3 (27.3%)	3 (12.5%)	8.795	0.118
	Klebsiella	5 (45.5%)	2 (12.5%)		
	Pseudomonas	1 (0.91%)	4 (24%)		
	Actinobacter	1 (0.91%)	2 (12.5%)		
	coagulase negative staph	0 (0%)	5 (31.3%)		
	Mixed organisms	1 (0.91%)	0 (0%)		
Bactria biofilm	Yes	10 (90.9%)	2 (12.5%)	16.231	0.000
	No	1 (9.1%)	14 (87.5%)		

Chi square test; *p* < 0.05 = significant, *ARDS* Acute respiratory distress syndrome, *MRSA* Methicillin-resistant Staphylococcus aureus

The serum levels of heat shock protein-70 and TNF-a were statistically significantly higher in subjects with pneumonia than in healthy controls, especially those who developed ARDS and those who had bacterial biofilm, as shown in Table 4.

**Table 4:** The association between bacterial biofilm formation and laboratory investigations of the studied population

Variable	Biofilm		No biofilm		Independent student t test/Mann Whitney U test	
	Mean	SD	Mean	SD	t/z	p-value
HB	10.37	1.19	10.25	0.81	0.299	0.768
HCT	31.18	3.34	30.71	2.43	0.401	0.693
WBCs	8.70	1.39	9.63	2.34	−1.288	0.210
Platelets	297.00	14.49	294.93	27.95	0.248	0.807
AST (U/L)	36.42	19.57	23.13	9.54	2.155	0.048
ALT (U/L)	41.75	22.05	30.00	6.60	1.783	0.099
Urea (mg/dl)	25.95	2.42	25.11	3.13	0.783	0.441
Creat (mg/dl)	0.70	0.08	0.76	0.13	−1.552	0.134
Na (mmol/L)	133.58	2.94	134.53	4.72	−0.64	0.528
K (mmol/L)	3.39	0.40	3.48	0.31	−0.681	0.504
Ca (mg/dl)	9.70	0.08	9.76	0.13	−1.552	0.134
PH	7.30	0.09	7.38	0.06	−2.743	0.013
PCO2 (mmHg)	44.25	8.86	41.93	6.10	0.771	0.450
PO2 (mmHg)	63.79	10.65	79	25.99	−1.717	0.099
HCO3 mEq/L	20.78	2.07	24.11	3.91	−2.843	0.009
PaO2/FiO2	127.58	31.58	316	55.65	−3.066	0.005
O2sat (%)	92.92	2.02	92.33	2.09	0.734	0.470
ESR (mm/hr)	56.33	13.95	43.73	15.36	2.229	0.035
CRP (mg/dl)	83.25	32.33	35.60	30.30	3.913	0.001

Independent student t test and Mann Whitney U test; *p* < 0.05 = significance

*SD* Standard deviation, *HR* Heart rate, *RR* Respiratory rate, *Temp* Temperature, *Hb* Hemoglobin, *HCT* Hematocrit value, *WBCs* White blood cells, *AST* Aspartate transaminase, *ALT* Alanine transaminase, *Creat* Creatinine, *Na* Sodium, *K* Potassium, *Ca* Calcium, *PO2* Partial pressure of Oxygen, *PCO2* Partial pressure of carbon dioxide, *HCO3* Bicarbonate, *PaO2/FiO2* Partial pressure of oxygen in arterial blood, fraction of inspired oxygen ratio, *O2 sat* Oxygen saturation, *CRP* C-reactive protein, *ESR* Erythrocytic sedimentation rate

At cutoff point of 5.5 ng/ml, the serum level of heat shock protein 70 (HSP70) had 93.3% sensitivity and 91.1% specificity. At a cutoff point of 442 pg/ml, TNF-a had 93.3% sensitivity and 86.7% specificity to predict ARDS in children with community-acquired pneumonia, as shown in Table 5.

**Table 5:** Comparison of HSP-70 and TNF-α levels in the studied groups according to ARDS and biofilm formation

	HSP-70 (ng/ml)		ARDS Vs. no ARDS	ARDS Vs. control	No ARDS Vs. control
	Mean	SD			
Pneumonia complicated by ARDS	6.35	1.82	0.004	<0.0001	<0.0001
Pneumonia without ARDS	4.05	1.88			
Healthy control	1.23	0.92			
P-value	<0.0001				

	HSP- 70 (ng/ml)		Biofilm vs no biofilm	Biofilm vs control	No biofilm vs control
	Mean	SD			
Biofilm	7.29	1.33	<0.0001	<0.0001	<0.0001
No biofilm	3.85	1.42			
Healthy control	1.23	0.92			
P-value	<0.0001				

	TNF-α (pg/ml)		ARDS Vs. no ARDS	ARDS Vs. control	No ARDS Vs. control
	Mean	SD			
Pneumonia with ARDS	505.75	141.65	0.003	<0.0001	<0.0001
Pneumonia without ARDS	315.73	154.09			
Healthy control	111.04	53.42			
P-value	<0.0001				

	TNF-α (pg/ml)		Biofilm Vs. no biofilm	Biofilm Vs. control	No biofilm Vs. control
	Mean	SD			
Biofilm formation	593.67	126.77	<0.0001	<0.0001	<0.0001
No biofilm formation	318.49	138.27			
Healthy control	111.04	53.42			
P-value	<0.0001				

ANOVA test: One way Analysis of Variance test; Multiple comparison between groups through Post Hoc test: Tukey's test, p <0.05 is significant

*ARDS* Acute respiratory distress syndrome, *TNF-α* Tumour necrosis factor-alpha, *HSP-70* Heat shock protein70

**Fig. 1:**
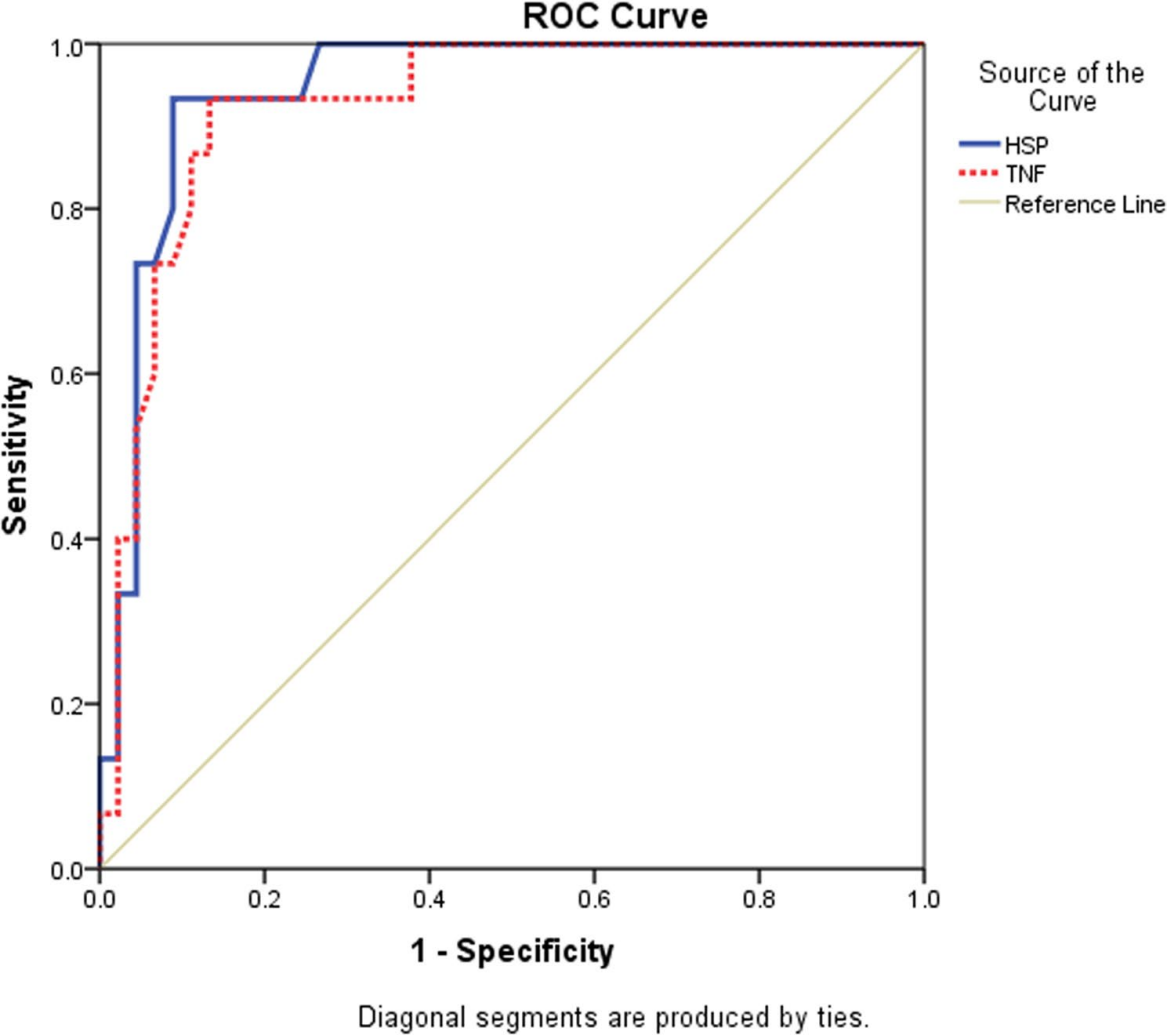
Roc curve for sensitivity and specificity of HSP-70 and TNF-alpha as predictors of ARDs in children with pneumonia

Risk factors for development of ARDS included elevated serum level of HSP-70 >5.5 ng/ml (OR:10.5; 95% CI: 4.081–27.018), elevated TNF-a >442 pg/ml (OR: 6; 95% CI: 2.997–12.011.997.011), bacterial biofilm formation (OR: 7.273; 95% CI; 1.983–26.948.983.948), respiratory acidosis (OR:6; 95%CI:2.438–14.766.438.766), and low PaO2/FiO2 <300 (OR:1.667; 95% CI:1.313–2.116.313.116) as shown in Table 6. (Fig. 1)

**Table 6:** The value of HSP-70 and TNF-alpha as predictors of ARDs in children with pneumonia

Biomarker	Cut off point	AUC	S.E	sensitivity%	specificity %	95% CI	
						Lower Bound	Upper Bound
HSP-70 (ng/ml)	> 5.5	0.944	0.029	93.30%	91.10%	0.886	1.000
TNF- α (pg/ml)	> 442	0.926	0.035	93.30%	86.70%	0.857	0.995

*ROC test* Receiver Operating Characteristic Curve, *AUC* Area under the Curve, *SE* Standard error, *C.I* Confidence interval, *HSP-70*: Heat shock protein-70, *TNF- α* Tumors necrosis factor-alpha

## Discussion

Pneumonia remains one of the globally leading causes of pediatric morbidity and mortality in children. New strategies for early diagnosis and successful treatment can be developed by identifying the risk factors associated with the emergence of complicated pneumonia [12, 13]. Recent information about the epidemiology, etiology, pathogenesis, treatment, and prevention of pediatric pneumonia has led to updates in several international and local guidelines; however, significant issues still require standardization [14]. Increased lung vascular permeability is a hallmark of ARDS, a severe type of inflammatory lung injury. Clinical indicators of ARDS include severe hypoxemia and bilateral opacities on chest imaging that cannot be attributed to cardiac failure or volume overload [15].

In our study 15 out of 60 children developed ARDS (25%), exhibiting higher heart and respiratory rates. Additionally, the PaO2/FiO2 ratio was low, indicating a ventilation-perfusion mismatch, nearly identical to that found by Choi et al. in their patients with severe pneumonia requiring intensive care unit admission [15].

In our study, we found that patients who developed ARDS had significantly higher rates of isolated bacteria and greater biofilm formation compared to pneumonia without ARDS (73.3% vs. 35.6% and 90.9% vs. 12.5%, respectively). The most commonly identified organisms were Klebsiella (11.66%), MRSA (10 %), Pseudomonas (8.3%), Actinobacter (5%), coagulase-negative staphylococci (8.3%), and mixed organisms (1.66%). This distribution is atypical for community-acquired pneumonia in children that can be explained as all our included children were referred for admission in pediatric intensive care unit after worsening of their symptoms despite medical treatment so their pathogens could be acquired nosocomial while receiving medical care before their referral to our center.

Patients with isolated bacteria exhibiting biofilm formation had significantly higher AST levels, acute phase reactants, and lower pH, HCO3, and PaO2/FiO2, suggesting greater severity of inflammation and worse ventilation. Yun et al. reported that pathogens were detected in 81% of cases (with pyogenic bacteria in 7%, atypical bacteria such as Mycoplasma pneumonia in 1%, and viruses in 73%) [16]. Additionally, another multi-country case-control study found that viruses were less common and bacteria more prevalent in very severe pneumonia compared to severe cases, with RSV having the highest etiological fraction among all pathogens [17].

In both prokaryotic and eukaryotic organisms, heat shock proteins (HSPs) are widely distributed and conserved protein families that maintain cellular proteostasis and protect cells from external stimuli. The molecular weights of HSPs determine their classification. As cellular molecular chaperones, they assist in the folding of newly synthesized polypeptides, the refolding of metastable proteins, the assembly of protein complexes, the dissociation of protein aggregates, and the degradation of misfolded proteins as part of an integrated network [18]. HSPs are also crucial for regulating the cell cycle, apoptosis, and cell signaling transduction. Thus, many diseases are linked to HSP malfunction [19].

Chen et al., found that heat shock proteins are related to many diseases, including cancer, neurodegeneration, and others [18]. In our study, serum levels of heat shock protein-70 were significantly higher in subjects with pneumonia compared to healthy controls, particularly in those who developed ARDS and in those with bacterial biofilm, showing 93.3% sensitivity and 91.1% specificity at a value >5.5 ng/ml.

TNF-α is a multifunctional Th-1 cytokine and one of the most significant inflammatory cytokines [20]. In our study, TNF-α levels were significantly higher in patients with pneumonia, particularly in those with ARDS, compared to healthy controls, demonstrating 93.3% sensitivity and 86.7% specificity at a threshold of>442 pg/ml for predicting ARDS in children with community-acquired pneumonia. Consistent with our findings, Bacci et al. reported that median TNF-a levels were higher in patients with severe RDS requiring mechanical ventilation [21]. In comparison with established biomarkers in children with community - acquired pneumonia (e.g. procalcitonin, IL-6), procalcitonin (PCT) showed 86% sensitivity and significantly higher specificity (50–90%) compared to CRP, IL-6, and WBC, making it a better discriminator of bacterial causes [22]. IL-6 is significantly elevated and associates with mortality, it offers high sensitivity (80–100%) but variable specificity (30–80%) making it better for rolling out ARDS than confirming it [23]. On the other hand, TNF-a is highly sensitive (due to early rise in inflammation) but inconsistent specificity, limiting standalone use. HSP-70 shows promise in prognostic stratification, but awaits further validation for routine predictive use. To improve patient assessment and care, the right biomarkers should be chosen based on the particular clinical situation [24].

Limitations of the study: our study has some limitations including: the small sample size, the cross-sectional design in addition to the single-center nature of the study, which may limit generalizability. Another limitation is lacking of viral pathogen evaluation among our included children and the inability to accurately define if the cause of pneumonia was community acquired or nosocomial. Despite that diagnosis of ARDS is based on OI but it cannot be calculated or compared to children who did not develop ARDS or control group as none of them was put on mechanical ventilation. Also we did not follow up serum level of Hsp-70 at the beginning and during mechanical ventilation.

## Conclusion

In children with pneumonia, the development of ARDS is associated with elevated serum levels of heat shock protein-70, elevated TNF-alpha, bacterial biofilm formation, respiratory acidosis, and a low Pa O2/FiO2 ratio. Moreover, HSP70 and TNF-a have good predictive values for ARDS in children with pneumonia. Assessment of such inflammatory markers at admission allows risk stratification and adjusts management strategies for children with pneumonia to prevent the development of ARDS [25]. 

## Data Availability

Data is provided within the manuscript.

## References

[1] Ki W. Yun-community-acquired pneumonia in children: updated perspectives on its etiology, diagnosis and treatment. Clin Exp Pediatr. 2024;67(2):80–9. 37321577 10.3345/cep.2022.01452 PMC10839192

[2] Matthay MA, Zemans RL, Zimmerman GA. Acute respiratory distress syndrome. Nat Rev Dis Primers. 2019;5(1):18. 30872586 10.1038/s41572-019-0069-0 PMC6709677

[3] Jain S, Williams DJ, Ampofo Kj AHSR, Bramley AM. Reedc,et al Community-acquired pneumonia requiring hospitalization among Us children. N Engl J Med. 2015;372:835–45. 25714161 10.1056/NEJMoa1405870 PMC4697461

[4] Jeon YH, Kim. JH-Treatment of community-acquired pneumonia in Korean children. Allergy Asthma Respire Dis. 2017;5:177–84.

[5] Evgen’ EV MB, Garbuz D.G, Zatsepina O-G. Heat Shock Proteins and whole Body Adaptation to Extreme Environments Springer; Berlin/Heidelberg,Germany: (eBook). 2014. 10.1007/978-94-017-9235-6.

[6] LubkowsKa A, Pluta w, StronsKa A, LalKo A.. Role of Heat Shock Proteins (HSP70 and HSP90) in Viral infection. Int J Mol Sci. 2021;22. 10.3390/ijms 22179366. 10.3390/ijms22179366 PMC8430838 34502274

[7] AFrazi A, Sodhi CP, Good M, Jia H, Siggers R, et al. Intracellular heat shock protein-of negatively regulates TLR4 Signaling in the new born intestinal epithelium. J Immunol. 2012;188:4543–57. 10.4049/jimmunol.1103114 PMC3331906 22461698

[8] Bordon JMJ, Fernandez-Botran R, Wiemken T. Lyetal bacteremia Pneumococcal pneumonia: clinical outcomes and preliminary results of inflammatory response. Infection. 2015;43(6):729–38. 26424683 10.1007/s15010-015-0837-z

[9] Teng NY. Detection of TNF-a,lL-6,IL-8 levels in serum of children with Mycoplasma pneumonia and its clinical significance-Chinese. J Nosocom Logy. 2012;22:529533–535.

[10] Yildizdas D, Aslan N. Pediatric acute respiratory distress syndrome updates in the light of the PALICC-2 guidelines. Turk Arch Pediatr. 2025;60(4):362–71. 40637326 10.5152/TurkArchPediatr.2025.24331 PMC12257685

[11] Freeman DJ, Falkiner FR, Keane CT. New method for detecting slime production by coagulase negative Staphylococci. J Clin Pathol. 1989;42(8):872–4. 2475530 10.1136/jcp.42.8.872 PMC1142068

[12] Gokcen DT, Beste 0. IremT, Betul P-. Aslinur o.,Guzinec: Risk factors for complicated Community-acquired pneumonia in children. Pediatr Int. 2022;64(1):e15386. 10.1111/ped.15386 36225107

[13] Yavuz S, Sherif A, Amirrad M, Sabet K, Hassan M, Abuelreish M, et al. A retrospective chart review of pediatric complicated community-acquired pneumonia: an experience in the al Qassimi women and children. Hosp Cureus. 2022;14(11):e31119. 10.7759/cureus.31119 PMC9636560 36382327

[14] Qiang Qin, Kun-ling Shen. Community-acquired Pneumonia and its Complications. Indian J Pediatr.2015.Aug; 82(8):745–sJ. 10.1007/s12098-015-1785-4 25976616

[15] Force ADT, Ranieri V-M. Ruben Feild GD. Acute respiratory distress syndrome: the Berlin Definition- JAMA. 2012;307:2526–33. 10.1001/jama.2012.5669 22797452

[16] Choi SH, Hong SB. Ko G.B. Viral infection in patients with severe pneumonia requiring intensive care unit admission. Am J Respir Crit Care Med. 2012;186:325–32. 22700859 10.1164/rccm.201112-2240OC

[17] Yun KW, Wallihan R, Desai A, Alter S, Ambroggio L, Cohen DM, et al. Clinical characteristics and etiology of community- acquired pneumonia in US children, 2015–2018. Pediatr. Infect Dis J. 2022;41:381–7. 10.1097/INF.0000000000003475 35143427

[18] Yun KW, Wallihan R, Desai A, Alter S, Ambroggio L, Cohen DM, et al. Multi-country case-control studies: Pneumonia Etiology Research for child Heath (PERCH), Study group of severe pneumonia requiring hospital admission. In children without HIV infection from Africa and Asia. Lancet. 2019:394:757–79. 10.1016/S0140-6736(19)30721-4 PMC6727070 31257127

[19] Chen H, JingYang Z, Qi H, Wu R, Wang, et al. Heat shock Proteins: Biological functions, pathological Roles, and therapeutic opportunities. Med Comm. 2022;3(3):e161. 10.1002/mco2.161 PMC9345296 35928554

[20] Edkins AL, Boshoff A. Geneal structural and functional features of molecular chaperones. Adv Exp Med Biol. 2021;1340:11–73. 34569020 10.1007/978-3-030-78397-6_2

[21] Teepe J, Grigoryan L, Verheij TJ. Determinants of community-acquired pneumonia in children and young adults in primary care. Eur Respir J. 2010;35:1113–7. 10.1183/09031936.00101509 20436174

[22] MR, Bacci CRP, Leme NPC, Zing N, Murad F, Adami, et al. IL-6 and TNF-a Senun levels are associated with early death in community-acquired pneumonia patients. Braz J Med Bio Res. 2015;48(5):427–32. 10.1590/1414-431X20144402 PMC4445666 25714883

[23] Moulin F, Raymond J, Lorrot M, Marc E, Coste J, et al. Procalcitonin in children admitted to hospital with community acquired pneumonia. Arch Dis Child. 2001;84(4):332–6. 10.1136/adc.84.4.332 PMC1718706 11259234

[24] Monique M, Matthew P, Hector R, Daniel M, Scott E, et al. Biomarkers associated with mortality in pediatric patients with cardiac arrest and ARDS. Resuscitation. 2021;170:184–93. 10.1016/j.resuscitation.2021.11.036 PMC8799511 34871756

[25] Yan Y, Hu Y, Wang X, Yu Z, et al. The predictive prognostic values of serum interleukin-z, interleukin-6, interleukin-8, TNF- α, and procalcitonin in surgical intensive care unit patients. Ann Transl Med. 2021;9(1):56. 33553349 10.21037/atm-20-6608 PMC7859771

